# Can GM soybean be reliably quantified after screening? A risk-based approach for optimizing GMO testing workflow

**DOI:** 10.1080/21645698.2026.2653897

**Published:** 2026-04-05

**Authors:** Davide La Rocca, Katia Spinella, Pietro Franceschi, Daniela Verginelli, Sara Ciuffa, Marisa Misto, Cinzia Quarchioni, Cristiana Fusco, Stefania Peddis, Pamela Bonini, Lorella Peroni, Ugo Marchesi

**Affiliations:** a National Reference Laboratory for GM Food and Feed, GMO Unit, Istituto Zooprofilattico Sperimentale del Lazio e della Toscana “Mariano Aleandri”, Rome, Italy; b Research and Innovation Centre, Fondazione Edmund Mach, San Michele all’Adige, Italy

**Keywords:** Cut–off value, GMO, qPCR, screening elements

## Abstract

GMO testing laboratories operating within the current European Union (EU) regulatory framework governing the presence of genetically modified organisms (GMOs) in food and feed (EC Reg. 1829/2003 and EC Reg. 1830/2003) perform a stepwise workflow from DNA extraction to quantification of genetically modified events. A very valuable intermediate step in guiding and optimizing this workflow is the screening phase, where any positives require the laboratory to proceed to the subsequent identification and quantification steps. Very often, however, samples with low GM content result positive at screening but then the GM component is not quantifiable, wasting time and resources. In order to overcome this issue, in this study a statistical framework was developed to predict the presence of soybean GM events based on the difference between the quantification cycle (Cq, also known as threshold cycle (Ct) of the Real time PCR technique) values observed from screening elements as cauliflower mosaic virus 35S promoter (P35S), the nopaline synthase terminator (T-nos), the 5-enolpyruvylshikimate −3-phosphate synthase gene (CP4 epsps) and *lectin* reference gene (Lec) (ΔCq values). The feasibility of this approach was successfully in-house verified on real life and spiked samples. This approach can be seen as a proof of concept to suggest how to optimize, on a statistical basis, the workflow of GMO testing laboratories that need to evaluate sample compliance with quantitative tests.

## 1. Introduction

A genetically modified organism (GMO) is subject to varying regulatory approaches in different countries around the world. Among countries with more restrictive legal framework, the European Union (EU), besides requiring the authorization of every GM event that wants to be introduced into the Union market, imposes the compulsory labeling on food and feed containing authorized GM events, if more than 0.9% GM component is present in the food/feed ingredient.^1–4^ The unique exception being feed matrices in which contamination by GMOs for which the authorization in EU is pending or expired is tolerated below 0.1% related to mass fraction of feed material.^5^

Official control laboratories must check compliance with labeling and traceability requirements for all authorized GM events in the EU. They must also check that unauthorized GM events do not enter the European market.^6^ Fortunately, the authorization procedure itself established by the regulations provides laboratories with reliable event-specific methods, based on polymerase chain reaction (PCR), for the identification and quantification of authorized and pending events (https://ec.europa.eu/food/food-feed-portal/screen/gmo/search). However, it would be unthinkable to analyze each individual sample by directly searching for all GM events using event-specific methods. Such an approach would be inefficient due to the ever-increasing number of GM events circulating on the market,^7^ and therefore to be searched for. Anyway, the direct detection and quantification of all known GM events is a costly and time-consuming activity.^8,9^ Moreover, without adopting screening methods, detection would be limited to known events. As a matter of fact, an optimized screening phase is generally very useful to decrease the number of event-specific methods to be performed afterward and to highlight the possible presence of some unknown events too. GMO control laboratories therefore adopt analytical strategies aimed at optimizing the analytical flow by introducing preliminary screening steps,^10,11^ whatever the screening elements chosen, that may differ from laboratory to laboratory, in the screening strategy adopted by the European Network of GMO Laboratories (ENGL) is based on the so-called “matrix approach.”^6^ In this way, GMO analysis in the EU context becomes a rather complex process essentially consisting of four phases as better described below. These phases can be considered self-supporting modules that can be independently validated.^12^ GMO analysis can therefore be defined as a “modular workflow”: the analytical method is indeed an organized series of steps, each involving the use of one or more modules which represent distinct tools or operations. For a single step, sometimes several alternative modules are available, allowing for increased flexibility.^13,14^ It’s clear that “modularity is necessary to allow for adaptation to the requirements of the different products”.^15,16^

In summary, the GMO detection workflow encompasses the following steps:

1. Detection of the ingredient(s)/component(s)/constituent(s) of the sample by means taxon-specific methods
2. GMO screening by means element and/or construct-specific methods
3. GM event identification by means qualitative event-specific methods
4. GM event quantification by means quantitative event-specific methods

According to the scheme described above, the GMO detection workflow is applied in the form of a decision support system (DSS) where detection/no detection events trigger (“Yes/No” result) the following course of action.^17–19^ The workflow starts with taxon-specific identification (step 1), in which the presence of a positive signal from PCR allows the transition to the next screening step (step 2). A negative screening result determines a compliant outcome (with the exception of events lacking of screening elements), while, in case of positivity to one or more screening elements, it is necessary to move on to the next phase (step 3) by searching for GM events compatible with the screening profile obtained. If any positivity is detected at the identification stage (step 3), if the positivity relates to an unauthorized GM event, the outcome of the analysis is noncompliance. If instead the positivity concerns authorized GM events or GM events for which an authorization procedure is pending or the authorization of which is expired, then quantitative tests are needed (step 4). After quantification, the sample is compliant if the GM percentage (considering the lower limit of measurement uncertainty) meets the legal limits.

Taxon-specific methods target species/taxon-specific DNA sequences.^13,20^ This strategy identifies and quantifies ingredients resulting from a species/taxon.^20^ The quantified value of species/taxon material permits the assessment of the relative GMO content per ingredient ^21^ and, as recommended by European commission, it is expressed as GM mass fraction (art 23, art 8).^22,23^

Screening methods target DNA sequences commonly included in gene constructs found in several transformation events ^20^ and for this reason, they can be used to determine whether a sample is likely to contain GMO or not. In addition, the screening profile of the sample helps to restrict GMO candidates by streamlining the following identification step. The screening strategy is based on specific PCR modules for detecting individual elements of the GM construct (promoters, terminators, coding sequences and selection markers) taken from viruses, bacteria, or other plant species.

Several real-time PCR screening methods for GMO testing have already been developed and validated so far. Among these, the most adopted screening methods are those for detecting the Cauliflower Mosaic Virus 35S promoter (P35S),^24,25^ the Nopaline synthase terminator (T-nos)^26,27^ and the 5-enolpyruvylshikimate-3-phosphate synthase gene (CP4 epsps).^28,29^

Nevertheless, many GM events, which do not contain any of the known screening elements, need to be detected directly through the event-specific methods, thus overcoming the screening phase.

The first screening methods described in literature were singleplex, but nowadays various multiplex system methods combining two or more targets in one reaction have been developed.^10,30–35^ Screening phase importance is demonstrated by its steering role for the subsequent analytical steps toward a subset of targets depending on the results of the screening tests. The interpretation of screening results is typically based on a two-dimensional matrix plotting, on one axis, selected detection methods and, on the other, selected GM events containing or not the screening elements sought.^6^ Traditionally, each laboratory uses its own matrix according to its screening strategy. However, in recent years, both the EURL-GMFF and other initiatives with GMO-relevant genome sequence databases, as EUginius, have proposed standardized web-based matrices that can be used online by any laboratory adopting the same methods.^6,18,36,37^

However, whilst the sensitivity of screening methods helps to detect even the faintest traces of GMOs, it also forces the laboratory to continue with the workflow to investigate the causes of a positive result, even when the signal is weak and late.

**Figure 1.**
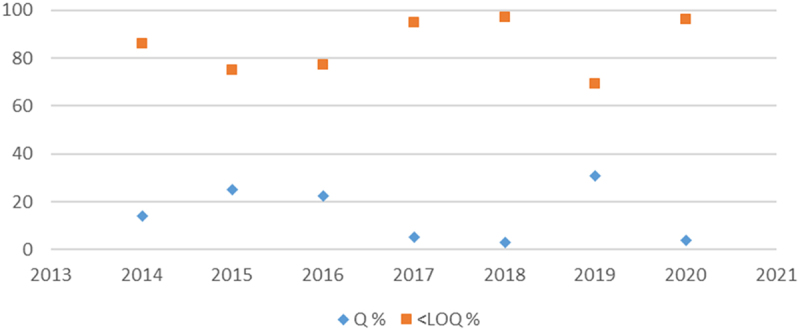
Percentage of outcomes of the overall amount of food samples tested positive to GM events in Italian national control plans over seven years. Q % stands for the quantified samples whereas < LOQ % category involves below loq, LOD and even all samples that didn’t test positive for GM event detection.

**Figure 2.**
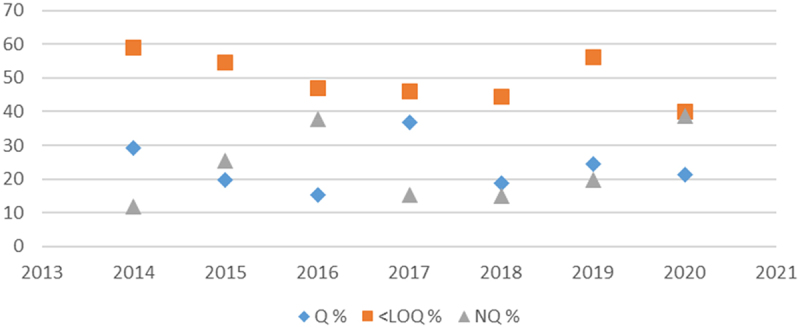
Percentage of outcomes of the overall amount of feed samples tested positive to GM events in Italian national control plans over seven years. Q % stands for quantified samples whereas < LOQ % category involves samples below loq and lastly NQ % category represents samples below LOD and those that didn’t test positive for GM event detection.

According to the results of the Italian control plans for the detection of GMOs in food and feed, it’s remarkable that in several samples that tested positive at the screening stage, the GMO content (% m/m) was often below the limit of quantification (LOQ) and sometimes below the limit of detection (LOD) (Figures 1 and 2). Indeed, the mean percentage of quantified positive GMO samples over the past seven years is 23.6% for feed and 14.9% for food (https://www.salute.gov.it/new/it/sezione/pubblicazioni/).

**Figure 3.**
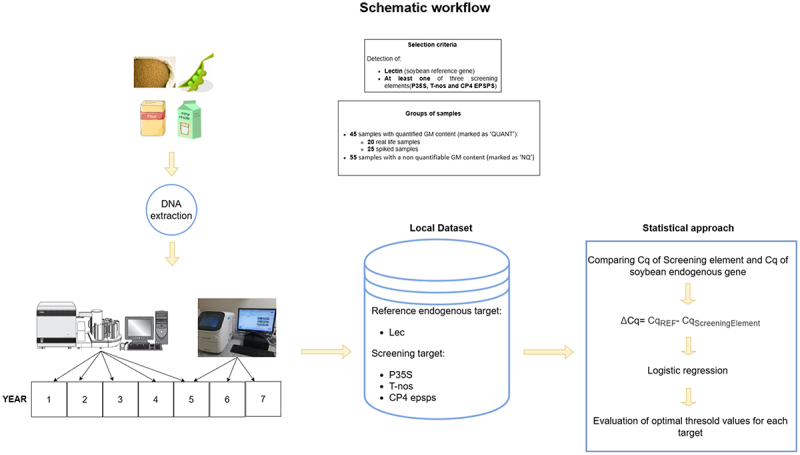
Schematic workflow of the study.

To address this issue, it was decided to analyze all available historical analytical data to see if it is possible to relate the instrumental output of positive screening tests to the quantifiability or non-quantifiability of the GM component of the sample. A dataset collected over seven years by the Italian National Reference Laboratory for GM food and feed (NRL) was then analyzed. The dataset was based on the results of routine GMO analyses performed on 100 samples: 71 samples were soybean-based matrices and the other 29 were matrices with soybean as ingredient in presence of other ingredients (maize, cotton, sugar beet, potato, rice, linen and rapeseed). The final dataset includes both real-life samples (previously analyzed by the NRL) and spiked samples with GTS 40–3-2 GM soybean Certified Reference Material (CRM) at different GM % levels. The choice of this GM soya event, which is widely used in the global agri-food market, as an experimental model comes from the fact that this transformation event includes P35S, T-nos and CP4 epsps, the three most popular screening targets in GMO testing laboratories. This gives the possibility of relating in the same sample the instrumental signal obtained for the soybean reference gene (*lectin*) and the corresponding instrumental signal obtained for each of the three screening elements in order to predict the outcome of the subsequent quantitative event-specific test. Specifically, since they are real time PCR tests, we examined the difference between the Cq (quantification cycle) value of each screening element and the Cq value of lectin gene, defined as delta Cq value (ΔCq) to identify consistent cutoff values able to direct the GMO downstream analytical pipeline. The detailed experimental design and statistical approach is outlined in Figure 3.

Notably, we have shown that these cutoff values can be reliably used to classify samples in which the GM event is below the LOQ value as non-quantifiable, allowing early establishment of sample compliance. In this study, samples are classified as “NQ” if at least one of the screening elements searched (as P35S, T-nos, CP4 epsps) is detected including both the case where GM events are measured by qPCR and are below LOD or LOQ and that one where no GM event is detected. As a matter of fact, the laborious and time-consuming process results in a huge number of detected GM events that are below the LOQ value. In order to validate the statistical method, we created spiked samples (spiked with different % levels of GTS 40–3-2 Certified Reference Material (CRM)) thereby confirming the reliability of the approach at classifying correctly between the two kinds of classes. The proposed workflow does not have the scope to substitute the official control for GMO detection, but it is thought of as an internal decisional-tool for agri-food industry authorities whose application can involve internal audits, HACCP procedures and the selection of GMO free products or with traces of GMO. This algorithm could be useful to assess the risk and simultaneously with other tools, such as search engines and databases, to search for GM events and screening elements.

Genetically modified (GM) crops are developed and cultivated worldwide, providing protection against insects and diseases, or tolerance to herbicides. *Glycine max* was taken into account as a model since it is one of the GM crops more cultivated globally,^38^ considering that in 2023 soybean has been the most widely planted species over 100.9 million hectares.^7^ Soybean has been currently reported to have 56 GM events authorized,^39^ being therefore the most representative crop in terms of number of samples analyzed by official control laboratories. In the past few years, numerous efforts have been made to optimize routine laboratory testing. For instance, Angers-Lousteau et al.^18^ developed JRC GMO-Matrix which is a precious instrument to plan and evaluate GMO screening strategies. In addition, Bohanec M. et al. ^40^ implemented SIGMO, a decision support system which assists especially producers involved in the European market for the assessment of GMO presence in food/feed products. The application field of these tools also includes NGS experiments. Indeed, Hurel J et al. ^41^ implemented a pipeline defined DUGMO which detects potential exogenous of coding sequences (CDS) insertion, CDS truncation or fusion with exogenous CDS of unknown genetically modified bacteria from high-throughput sequencing data, using a large number of classification methods, within a machine learning procedure. Table S1 resumes many of the available worthwhile tools and frameworks useful for supporting GMO laboratories in their activities. Nevertheless, none of the above mentioned solutions considered the usage of ΔCq values for streamlining the GMO testing workflow.

## 2. Materials and Methods

### 2.1. Selection of Routine Samples and Preparation of Spiked Samples

Based on historical routine analytical data analyzed over seven years, samples containing soybean and resulted positive for the detection of some representative screening elements (CP4 epsps, P35S and T-nos) were divided into two groups. The first group included 20 samples, marked as “QUANT,” for which a quantitative result to GM soybean events could subsequently be obtained (quantifiable samples). The second group included 55 samples, called “NQ,” for which neither a quantitative result to GM soybean events could subsequently be obtained (below LOQ, below LOD) nor any GM events could be detected. Moreover, to increase the representatives of the first group, a total of 25 spiked samples at five different GM soybean levels (0.1, 0.5, 1, 2 and 5%) was achieved by mingling a positive material (100% GM) of CRM GTS 40–3-2 with non-GM material as described in Hougs et al. (2017 annex 3).^42^ Specific amounts of CRM GTS 40–3-2 soybean 10% were added into negative routine samples containing soybean considering the concentration in the absolute copy number of the reference gene *lectin* (Lec) measured by real time PCR. The routine samples used for spiked samples preparation were previously analyzed for GMO detection and included a range of soybean-based samples with different composition, processing level and GM content.

The certified reference material (CRM) GTS 40–3-2 10% soybean (ERM-BF410 ep) and non-modified soybean (ERM-BF410ap) was purchased from the European Commission, Joint Research Centre Directorate F – Health, Consumers and Reference Materials (Retieseweg 111 B-2440 Geel, Belgium). All the samples are recorded in Table S2.

### 2.2. DNA Extraction and Assessment of DNA Purity

For the first five years the DNA extraction had been performed with the Generon ION Force DNA Estractor FAST (©Generon S.p.A, Italy) and later with the RSC PureFood GMO by using an extractor Maxwell®RSC Instrument (Promega Madison, WI, USA), according to the manufacturer’s instructions. As a test portion, 2 g of real-life samples and 0.15 g of soybean CRMs were used for extracting DNA in two replicates (respectively 2 and 0.15 g each replicate). The concentration and purity of the extracted DNA were evaluated by spectrophotometric measurements (Eppendorf BioPhotometer) at 260 nm, 260/280 ratio, and 260/230 ratio according to the ISO 21570:2005 ^43^ and to the manufacturer’s instructions. The quality of the extracted DNA was assessed by real time PCR performing an inhibition test of the *lectin* reference gene carried out in two dilution levels, each level measured in duplicate as described in Hougs et al. 2017 annex 2 JRC Technical Report.^42^ As reported in Verginelli et al., ^43^ both the extraction methods used in this study are comparable when taking into account food and feed samples with the exception of challenging matrices.

### 2.3. Real Time PCR Assays

Real time PCR assays were made by using two instruments: ABI Prism 7900HT Fast Real-Time PCR System (Applied Biosystems®, Foster City, CA, USA) until the half of the fifth year and after, QuantStudio™ 7 Flex Real-Time PCR System (Life Technologies, Foster City, CA, USA).

The following thermal profile was used for PCR amplification for both instruments: UNG pre-treatment of 2 min at 50°C, initial activation of the polymerase for 10 min at 95°C followed by 50 cycles of 95°C for 15 s and 60°C for 60 s. The Real Time PCR reactions of P35S and CP4 epsps methods were set up with a ready to use TaqMan® Universal Master Mix or TaqManTM Universal Master Mix II with UNG (2x) (Applied Biosystems®, Foster City, CA, USA). However, the T-nos Real Time PCR reaction involved 2x TaqManTM Gene Expression Master Mix and the Universal Master Mix II with UNG (2x) (Applied Biosystems®, Foster City, CA, USA) until the first half of the fifth year and from the second half of the fifth year, respectively. The primers and probes for real time PCR were supplied by Eurofins genomics (Ebersberg, Germany). The sequences of primers and probes for the endogenous gene, screening elements and GTS 40–3-2 are listed in Table 1. The amplification was performed in a final volume of 25 µl with a final DNA amount of 100 ng. Results were analyzed using the SDS Standard Core (Applied Biosystems®, Foster City, CA, USA) and QuantStudio™ 7 Real-Time PCR Software (Life Technologies, Foster City, CA, USA), for ABI Prism 7900HT Fast Real-Time PCR System and QuantStudio™ 7 Flex Real-Time PCR System, respectively. The baseline and the fluorescence threshold parameters were automatically set up by the instruments.

**Table 1.** Overview of real-time PCR methods used in this study.

Primer and probe	Gene name, amplicon lenght and 5‘−3’ Sequence	Final concentration	Reference
	***LECTIN*** **(LEC) 74 bp**		44
Lec-F	5’- CCA GCT TCG CCG CTT CCT TC −3’	0.3 pmol/μl	
Lec-R	5’- GAA GGC AAG CCC ATC TGC AAG CC −3’	0.3 pmol/μl	
Lec-P probe	FAM 5’-CTT CAC CTT CTA TGC CCC TGA CAC-3’TAMRA	0.1 pmol/μl	
	**Promoter P35S 84 bp**		24
35S-F3	5’- ATg CCT CTg CCg ACA gT −3’	0.4 pmol/μl	
35S-R	5’-AAg ACg Tgg TTg gAA CgT CTT C-3’	0.4 pmol/μl	
35S-TMP	FAM 5’-CAA AgA Tgg ACC CCC ACC CAC g-3’ TAMRA	0.2 pmol/μl	
	**Terminator T-nos 84 bp**		27
180 F	5’-CAT gTA ATg CAT gAC gTT ATT TAT g-3’	0.4 pmol/μl	
180 R	5’-TTg TTT TCT ATC gCg TAT TAA ATg T-3’	0.4 pmol/μl	
Tm-180	FAM 5’−ATg ggT TTT TAT gAT TAg AgT CCC gCA A 3’ (TAMRA)	0.1 pmol/μl	
	**Terminator T-nos 104 bp**		29
5F	5’-gTA ATg CAT gAC gTT ATT TAT gAg A-3’	0.3 pmol/μl	
4 R	5’-TAA TTT ATC CTA gTT TgC gCg C-3’	0.3 pmol/μl	
P1	FAM 5’-TgC ggg ACT CTA ATC ATA AAA ACC CA-3’ TAMRA	0.25 pmol/μl	
	**CP4 epsps 145 bp**		28
Sttmf3a	5’-gCA AAT CCT CTg gCC TTT CC-3’	0.6 pmol/μl	
Sttmr2a	5’-CTT gCC CgT ATT gAT gAC gTC-3’	0.6 pmol/μl	
Sttmpa	FAM 5’-TTC ATg TTC ggC ggT CTC gCg-3’ TAMRA	0.2 pmol/μl	
	**MON40-3–2 SOYBEAN 84 bp**		45
40–3-2 AF	5’- TTC ATT CAA AAT AAG ATC ATA CAT ACA GGT T- 3’	0.3 pmol/μl	
40–3-2 AR	5’GGC ATT TGT AGG AGC CAC CTT–3’	0.3 pmol/μl	
40–3-2 AP	6-FAM 5’-CCT TTT CCA TTT GGG − 3’ MGBNFQ	0.1 pmol/μl	

Each DNA replicate was analyzed in two PCR replicates for qualitative PCRs and in three PCR replicates for quantitative PCRs.

### 2.4. Analytical Framework

The analytical framework of the proposed methodology is shown in Fig. 3. It was developed to investigate the use of ΔCq values (CqREF – CqScreeningElement) in routine analysis of GMOs involving it in the decision-making of further steps as a supporting instrument. This approach aims to determine the samples that are potentially quantified with GM content of authorized GM events above the labeling threshold (0.9%) or the 0.1% minimum required performance limit (MRPL) according to Regulation (EU) No 619/2011.^5^ In our case study, we selected samples containing soybean that also included at least one screening element. A logistic regression was applied to the resultant ΔCq values in order to identify applicable threshold values at diverse confidence limits. Such different intervals are essential to comprehend the degree of error occurring in the classification of the computed cutoff. In addition, the use of “spiked” samples is central to the evaluation of the predictability of ΔCq thresholds. ΔCq values were obtained by calculating the difference between the Cq of the taxon-specific assay (i.e. CqREF) and the Cq of the screening element assay (i.e. CqScreeningElement).

### 2.5. Statistical Analysis

All the statistical analyses were performed in R,^46^ a language and environment for statistical computing and graphics, relying on the tidyverse package for data visualization and handling.^47^

A logistic regression approach was applied to the individual ΔCq of the three screening elements in order to identify consistent thresholds for the probability of a sample belonging to the “QUANT” class on the basis of its specific ΔCq.

The structure of the logistic regression model is shown in the following formula:

$$\ln\left(\frac{P\left(\mathrm{QUANT}\right)}{1-P\left(\mathrm{QUANT}\right)}\right)=\beta_{0}+\beta_{1}\cdot \Delta C_{t}$$

The validation of the model performance has been performed by applying a cross-validation scheme (5 folds, 10 times) and assessed with AUC. In addition, the validation employed on the real dataset has been compared with the one constructed on a dataset with randomly shuffled target categories

Spiked samples were included in the “QUANT” category, thereby reducing the unbalancing effect on the predictive potential.

The model structure indicates that the logarithm of the odd ratios is linear in the predictor and the equation shown below indicates how to obtain, from the model equation, thresholds corresponding to a given P(QUANT):

$$T_{P\left(\mathrm{QUANT}\right)}=\frac{\ln\left(\frac{P\left(\mathrm{QUANT}\right)}{1-P\left(\mathrm{QUANT}\right)}\right)-\beta_{0}}{\beta_{1}}$$

## 3. Results and Discussion

### 3.1. Δ. Cq Calculation and Evaluation Through the Samples

**Figure 4.**
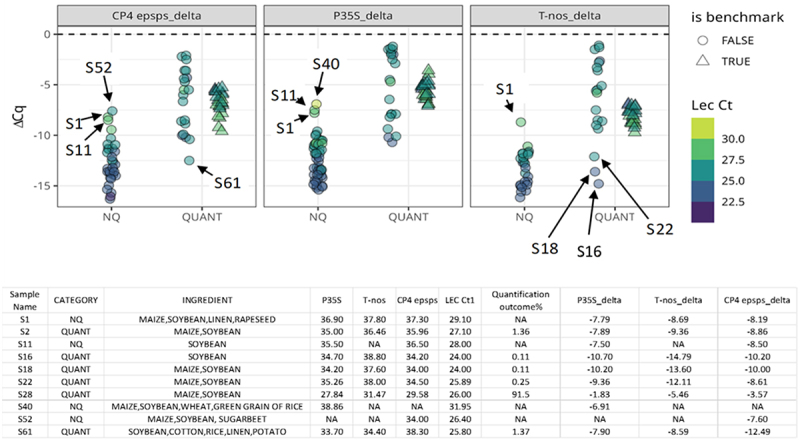
Raw ΔCq for the three screening elements in all the samples included in this study. The colour scale highlights the signal of the endogenous gene for soybean. Triangles describe the quantified spiked samples marked as “benchmark” group, whereas circles belong to the real life samples. Below the dotplot, a descriptive summary table of the samples that behave in an unusual manner is shown. ‘QUANT’ stands for quantified outcome whereas ‘NQ’ includes cases with GM events measured by qPCR and resulted below LOD or loq and cases where no GM event was detected.

Figure 4 displays the ΔCq values of the two groups (NQ and QUANT) in relation to each selected screening element. The spiked samples are classified as benchmark true and depicted as triangles, while the real-life samples are classified as benchmark false and displayed as circles. The shades of color depend on the Cq value of endogenous gene, Lec.

A general trend for all the screening elements considered is observed and, as expected, spiked samples (triangles) are more collapsed than the other groups. “QUANT” is the most variable group, showing few samples showing extreme behavior. Furthermore, group “NQ” showed some atypical samples too, that can occur depending on several aspects like the multi ingredient composition or the low DNA quality. Indeed, the sample S11 belongs to a challenging food matrix, a soy drink with a poor DNA quality and low yield. Sample S1 is a complex feed matrix composed of several plant species, where the GTS 40–3-2 event was below LOQ and a high Cq value of the endogenous *lectin* gene was reported (*lectin* Cq = 29.1). No issues in the DNA extraction have been observed, thereby signals of the screening elements detected could be explained by the presence in trace amounts of other GM events of other plant species whose analysis wasn’t required at the time. Eventually, samples S40 (with a high Cq value of the endogenous *lectin* gene) and S52 having only one screening target detected, do not reveal any specific issue in the steps preceding the quantification phase. In addition, both samples are complex feed composed of several plant species and the detected screening element could explain in this case, with trace amount of other GM events.

Regarding the “QUANT” group referring to real-life samples (circles), 4 uncommon samples were identified, three of these are observed in the T-nos plot and the last one in the CP4 epsps plot. Firstly, all the T-nos atypical samples (S16, S18 and S22) were analyzed with the Permingeat’s T-nos screening method ^29^ and the quantified event/s were respectively 0.11% (sum of GTS 40–3-2 and MON89788 not containing T-nos), 0.11% (GTS 40–3-2) and 0.25% (GTS 40–3-2), respectively. Moreover, other samples analyzed with the same T-nos method are S2 and S28 showing values of 1.36% (GTS 40–3-2) and 100% (sum of 80% GTS 40–3-2, 10% MON87701 and 10% MON89788) respectively. The reason could be that the previous screening method for T-nos ^26,29^ had a LOD of 9 haploid genome copies, higher than the LOD of the latest method used for the other samples (LOD of 4 haploid genome copies experimentally verified in our laboratory).^27^

Lastly, sample S61 shows for the CP4 epsps a lower ΔCq value than those of the other samples. This sample is a composite animal feed containing several ingredients, but only soybean (GTS 40–3-2 1.4% and 10.5% MON87708) and cotton (GHB119 0.86%) GM events were quantified. It must therefore be considered that two of the three above mentioned quantified GM events contain P35S and T-nos with the consequence of strengthening the signal for these two screening elements, while CP4 epsps is only present in GTS 40–3-2 soybean GM event. This is probably why CP4 epsps appears less represented in the specific plot for this screening target.

The spiked samples containing different GTS 40–3-2 levels, ranging from 0.1% to 5%, and represented by triangles in the Figure 4, approximately mimic an instrumental response without the contribution of the variability of a real-life sample and, as expected, show a similar distribution for each screening target. Interestingly, the whole group of P35S spiked samples gets closer to the higher part of the graph, ranging around −5 ΔCq value, while the CP4 epsps spiked group places below the −5 ΔCq value with few samples near −10 ΔCq, as well as the T-nos spiked group.

To sum up, for highly processed or heterogeneous matrices, often due to the poor DNA quality and low DNA quantity, the detection of the endogenous reference gene could be difficult or not optimal. In addition, sometimes, the simultaneous presence in the sample of different GM events (maybe from different plant species) containing the same screening target can lead to unclear results. Indeed, low quantities of many GM events often result in <LOQ values, making impossible to have detailed information about the actual GM content.

### 3.2. Evaluation of the Logistic Regression Analysis

Logistic regression models were (each for a screening target) built to establish how robust can be the relationships between the ΔCq values and the “QUANT” outcome, calculating three thresholds for three confidence intervals (99%, 97%, and 95%) for each target. The R^2^ McFadden values for the three models are 0.74, 0.63, 0.64 for P35S, T-nos and CP4 epsps, respectively. In addition, the validation of the model performance is shown in Table 2, where the AUC values of both the cross validation and the comparison with a shuffle dataset are calculated for each Δ

**Table 2.** Results in terms of AUC of the 5 folds cross-validation applied for the model perfomance. The second column represents values computed from the real dataset, whereas the third from the one with randomly shuffled target categories.

Element	Real mean AUC [95% CI]	Shuffled mean AUC [95% CI]
P35S_delta	0.982 [0.976, 0.988]	0.392 [0.371, 0.412]
T-nos_delta	0.953 [0.937, 0.969]	0.474 [0.430, 0.517]
CP4 epsps_delta	0.967 [0.958, 0.976]	0.448 [0.407, 0.489]

All the three models show a good performance for the cross-validation scheme, suggesting a large capacity to distinguish between “QUANT” and “NQ.” Furthermore, the unbalancing caused by the number of samples for each class (45 vs 55) shows a slight effect, as demonstrated by the performance of the logistic model on the data with shuffled class labels. Indeed, a little deviation from 0.5 is obtained.

**Figure 5.**
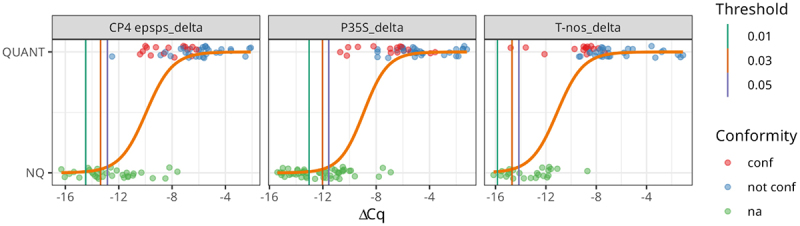
Graphical representation of the results of the logistic regression analysis on the individual ΔCq. Blue dots identify the measured values for the individual samples. The orange line shows the estimated probability of being quantified as a function of the ΔCq. The vertical bar highlights the position of the thresholds corresponding to probabilities of 99% (dark green vertical line), 97% (orange vertical line) and 95% (navy blue vertical line). In addition, the colour of the dot indicates the conformity or non-conformity of the sample to the labelling rules set by the EU, based on the GM content observed following its quantification. In particular, the ‘NQ’ category has not available data (na, green), while the ‘QUANT’ one can be classified as ‘conf’ (red) or ‘not conf’ (blue) if the GM content is below or above the 0.9% GM content, respectively. The GM content % used for the ‘conformity’ label is represented by the sum of soybean GM events that include at least one of the three screening targets considered in this study.

Figure 5 shows the relationship between our dependent variable (whether a sample belongs to the “QUANT” or “NQ” class) and the independent variable, in our case the ΔCq value, for each of the screening elements selected. Experimental evidence demonstrates fairly well that the ΔCq-based interpretation of the probability of a sample to be quantified can be valid, where possible, for the adoption of further steps in the GMO testing analytical workflow. Thresholds extrapolated from P35S, CP4 epsps and T-nos data are reported in Table 3 at three different confidence intervals (99%, 97%, and 95%). Looking at Table 3 and Figure 5, ΔCq thresholds for P35S are lower, in absolute value, than the others, moreover, for this screening element, no “QUANT” may be misclassified at all the three confidence intervals. Also for CP4 epsps, the “QUANT” group is correctly classified within ΔCq thresholds at all probability levels, but in this case the ΔCq value of sample S61 (in blue in Figure 5), is very close to the limit of the 95% confidence interval despite the fact that it is not only, of course, a quantifiable sample but also contains more than 0.9% of GM soybean (GTS 40–3-2), and therefore is non-compliant with the labeling rules applied in the EU. However, it must be emphasized that this sample, despite this close call on CP4 epsps, is by a broad margin correctly classified for the other two screening elements. Lastly, the T-nos “QUANT” group is fully correctly classified only at 99% confidence level while at 97% and 95% confidence level one sample is untypical (S16) (Figure 5). In any case, all samples found, after quantification, to be non-compliant with EU labeling rules were correctly classified as QUANT by the proposed statistical model, for all three confidence levels, in all three screening targets analyzed. What is shown in Figure 5 is that it is possible to predict the quantifiability of samples that test positive for screening, using the adopted model with the proposed thresholds, without the laboratory running the risk of missing non-compliant samples. In addition, looking at both the picture and the R^2^ values, the best model is represented by that of P35S.

**Table 3.** Δcq thresholds corresponding to different estimated probabilities of being classified as quantified (p_*quant*._).

Screening Element	*p*_*quant*_ = 0.01	*p*_*quant*_ = 0.03	*p*_*quant*_ = 0.05
P35S_delta	−13.02	−12.03	−11.55
T-nos_delta	−15.85	−14.69	−14.14
CP4 epsps_delta	−14.47	−13.37	−12.84

The results obtained therefore show that even a single screening target is able to represent a good predictive model for estimating the quantifiability of GM events containing that target in the sample.

However, each screening method has its own particularities in terms of performance, and therefore we found it useful to compare in pairs the ΔCq values obtained by the screening methods for the same samples.

**Figure 6.**
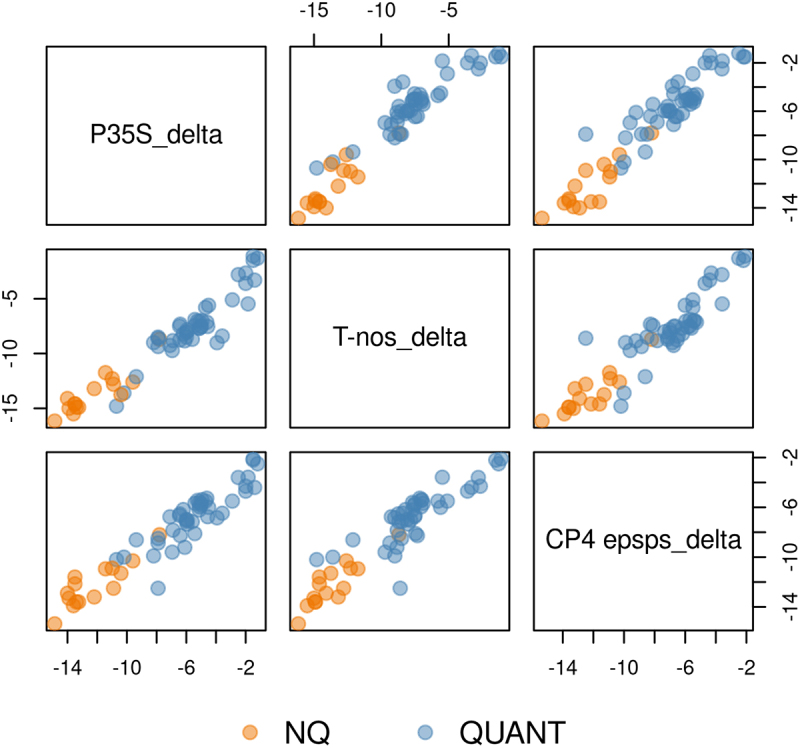
Pairs plot showing the associations between the measured ΔCq for the individual screening elements. Colours indicate the outcomes of the samples.

Figure 6 is a qualitative representation of pairwise comparisons of ΔCq values across the three screening targets. Except for a few deviations, all graphs show a good correlation with an almost linear distribution over the entire range of values. It’s clear that the simultaneous application of both ΔCqs of P35S and T-nos could be an effective prediction method for the outcome of samples.

Overall, it was observed that the approach based on estimated ΔCq thresholds is ideal for sample allocation to the “QUANT” category, and that the application of this support tool to more than one screening element can even more consistently allow a good number of samples to be excluded from subsequent quantification steps. It may be challenging when you have to deal with real-life samples which contain GM events from several GM plant species containing screening targets whose detection signal is associated with not only one GM event. This situation would result in a ΔCq compression that would artificially classify the sample among the QUANTs. Unfortunately, this problem cannot be solved unless all events in the sample are identified and quantified, since the screening data alone would not be sufficient in this case.

### 3.3. Spiked Samples

In order to validate our results and to evaluate whether the threshold can be applied to real-life samples, in which the soybean is a common ingredient, we made spiked samples as explained in the Materials and Methods section.

The average GM content for each real-life and spiked samples was calculated (Table 4). A slight variability of GM % was observed for the smallest amount of GM content added (Figure S1).

**Table 4.** Information about the matrix, ingredients, and the measured GM content for all the spiked samples at five different expected concentration levels (5%, 2%, 1%, 0.5%, 0.1%).

Matrix	Ingredients	MON 40–3-2 GM content%				
Animal feed	soybean, maize	4.30	3.06	1.14	0.62	0.34
Animal feed	soybean, maize	4.35	2.00	1.29	0.75	0.43
Flour	Soybean	4.61	2.44	1.42	1.23	0.53
semi processed food	Soybean	4.30	1.61	1.03	0.45	0.11
soybean	Soybean	4.96	1.94	1.45	0.79	0.31

## 4. Conclusions

The results obtained with the ΔCq cutoff approach proposed show that it is possible to predict with good accuracy whether the w/w GM content of a sample, for which we have data on screening and taxon-specific tests, will be quantifiable or not. This approach is a proof of concept aimed at reducing the number of samples that would have to be quantified due to positive screening results.

Obviously, in order to estimate these threshold values, and to be able to apply the predictive model proposed, the GMO testing laboratory will have to analyze a sufficient number of past analyses in the manner we have described. Indeed, the usage of spiked samples helped us to increase the number of QUANT samples with the further advantage of having a controlled scenario for them. What can influence estimates is mainly PCR efficiency, which can vary from method to method, also affected by the quality of the template DNA, which can vary depending on the food matrix and the extraction method used.^43^ In our experience, we saw that up to 95% confidence two of the three screening methods analyzed correctly classified all quantifiable samples. Only in one case (T-nos) and on one sample was this confidence level not sufficient and had to be increased to 99% to cover all the QUANT samples. It is therefore clear that by applying a threshold corresponding to a lower confidence percentage (95%), there is a greater reduction in the number of samples to be subsequently quantified while on the other hand there is a higher risk of losing some quantifiable samples. Therefore, the laboratory decides the risk-benefit ratio acceptable to it according to the context in which it operates. For example, an official control laboratory will not be able to afford any margin of error in this estimate unless the competent authority establishes otherwise, whereas a laboratory involved in own-checks programmes might agree with its customer on the acceptable risk level weighing the savings from the quantitative analyses not carried out and the costs resulting from any noncompliance. In the last decade, several research groups have proposed theoretical models that relate the instrumental output of real time PCR to the GMO content of the sample, also with the aid of control plasmids.^48–50^ Furthermore, the general idea of forecasting outcomes only considering the qualitative results through the evaluation of a difference between Real time PCR Cts has been explored also in other fields such as clinics and genetic diversity in Chen et al.,^51^ Bordoy et al.,^52^ Yoshioka et al.,^53^ and suggested as a valid decision support tool.

GM soybean is the most prevalent GM plant species in food and feed, so the good results obtained on this model optimistically open the way for further confirmatory investigations on other GM crops. Ideally, the implementation of this approach for different GM crops with a good number of GM screening targets at the same time could provide the GMO testing laboratory with a precious tool for streamlining the analysis of GMOs in food and feed.

## Supplementary Material

Supplemental Material

## Data Availability

The original contributions presented in this study are included in the supplementary material. Further inquiries can be directed to the corresponding author(s).
