# Supplementary material for: Can GM soybean be reliably quantified after screening? A risk-based approach for optimizing GMO testing workflow
Source: GM Crops Food. 2026 Apr 5;17(1):2653897. doi: 10.1080/21645698.2026.2653897 (PMC13051587; doi:10.1080/21645698.2026.2653897)
Supplement: Supplemental Material [file KGMC_A_2653897_SM8843.zip › Figure S1 GM content in Spiked Samples.docx]

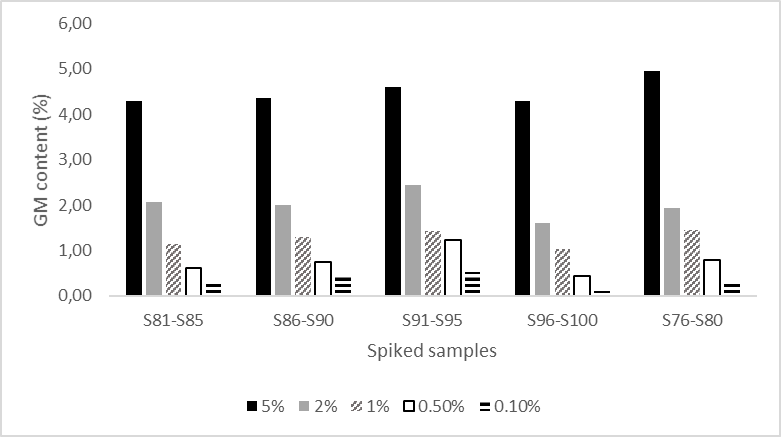


**Figure S1 Information about the GM content of GTS 40-3-2 expressed as percentage in the Spiked Samples performed in this study**
